# Spatio-temporal patterns of an anthrax outbreak in white-tailed deer, *Odocoileus virginanus,* and associated genetic diversity of *Bacillus anthracis*

**DOI:** 10.1186/s12898-015-0054-8

**Published:** 2015-12-15

**Authors:** Jocelyn C. Mullins, Matthew Van Ert, Ted Hadfield, Mikeljon P. Nikolich, Martin E. Hugh-Jones, Jason K. Blackburn

**Affiliations:** Department of Geography, Spatial Epidemiology and Ecology Research Laboratory, University of Florida, Gainesville, FL USA; Emerging Pathogens Institute, University of Florida, Gainesville, USA; Department of Emerging Bacterial Infections, Bacterial Diseases Branch, Walter Reed Army Institute of Research, Silver Spring, MD USA; Department of Environmental Sciences, School of the Coast and Environment, Louisiana State University, Baton Rouge, LA USA

**Keywords:** *Bacillus anthracis*, Anthrax, Wildlife, Genetic diversity, Outbreak, Transmission

## Abstract

**Background:**

Anthrax, a soil-borne zoonosis caused by the bacterium *Bacillus anthracis*, is enzootic in areas of North America with frequent outbreaks in west Texas. Despite a long history of study, pathogen transmission during natural outbreaks remains poorly understood. Here we combined case-level spatio-temporal analysis and high resolution genotyping to investigate anthrax transmission dynamics. Carcass locations from a single white-tailed deer, *Odocoileus virginanus,* outbreak were analyzed for spatial clustering using *K*-function analysis and directionality with trend surface analysis and the direction test.

**Results:**

The directionalities were compared to results of high resolution genotyping. The results of the spatial clustering analyses, combined with deer movement data, suggest anthrax transmission events occur within limited spatial areas, with carcass locations occurring within the activity space of adjacent cases. The directionality of the outbreak paralleled adjacent dry river beds. Isolates from the outbreak were represented by a single genotype based on multiple locus variable number tandem repeat analysis (MLVA); four sub-genotypes were identified using single nucleotide repeat (SNR) analysis.

**Conclusions:**

Areas of high transmission agreed spatially with areas of higher SNR genetic diversity; however, SNRs did not provide clear evidence of linear transmission. Overlap of case home ranges provides spatial and temporal support for localized transmission, which may include the role of necrophagous or hematophagous flies in outbreaks in this region. These results emphasize the need for active surveillance and prompt cleanup of anthrax carcasses to control anthrax both during outbreaks and between seasons.

**Electronic supplementary material:**

The online version of this article (doi:10.1186/s12898-015-0054-8) contains supplementary material, which is available to authorized users.

## Background

Anthrax, caused by the spore-forming, Gram positive bacterium *Bacillus anthracis*, is an enzootic or re-emerging disease of livestock and wildlife in the United States. Within enzootic areas, anthrax occurs sporadically in most years. In select years, large outbreaks occur in association with climatic conditions [1]. Among managed wildlife populations, anthrax outbreaks cause substantial economic losses through reduced income, loss of wildlife, and expenditures for surveillance, veterinary support, laboratory testing and carcass disposal (e.g. burning or burial). Additionally, the pathogen has the potential for spillover into livestock populations, with financial, agricultural, and public health consequences. Despite literature describing soil characteristics of anthrax zones [2–5] and empirical descriptions of anthrax occurrence in endemic areas [6–10], mechanisms that initiate and perpetuate outbreaks in nature remain poorly characterized.

In herbivores, infection with *B. anthracis* occurs through environmental pathways. After death of the animal, vegetative cells spilled from the carcass sporulate and contaminate the environment, thus becoming available to infect the next animal. Certain soil characteristics, such as high calcium content and higher alkalinity, may support spore persistence for long periods of time [5, 11]. Although an outbreak might result when all animals are independently exposed to an environmental source, potential transmission mechanisms which perpetuate an outbreak include contact with contamination in the environment around carcasses [12] or mechanical transmission by hematophagous flies [13, 14]. Hugh-Jones and de Vos [15] and Hampson et al. [9] suggested environmental transmission of *B. anthracis* likely differs based on the feeding habits of hosts during anthrax outbreaks. Grazers are likely to ingest spores or vegetative cells which are present on grasses or in soils due to runoff from rains or contamination from carcasses [10, 12]. Browsing species, in contrast, may consume vegetative cells or spores deposited on leaves by necrophagous flies [16, 17]. Because outbreaks are spatially localized and temporally limited across most ecosystems [9, 15], micro and meso scale ecological processes, driven by local climate, host and vector species, and host behavior, are important, but poorly studied, determinants of anthrax ecology. Characterizing these processes therefore informs prevention and control efforts, the latter of which rely on prompt identification and disposal of carcasses [18].

While several spatial studies have examined spatial and spatio-temporal patterns of outbreaks from the national (livestock surveillance data) [19, 20], regional (multiple farm epizootic) [21] and local (locations of carcasses over several years) [5] scale, fewer studies have examined spatio-temporal outbreak dynamics at the level of the individual carcass within a single outbreak [13]. Furthermore, few studies have linked these analyses to the genetic diversity of *B. anthracis* within the outbreak. Combining genetic information with spatio-temporal analyses could be a powerful method to explaining anthrax outbreak dynamics and generating hypotheses about environmental transmission mechanisms. Broadly, molecular diversity of *B. anthracis* populations can be described using three sets of molecular markers. Canonical single nucleotide polymorphisms (canSNPs) place bacterial isolates into broad phylogenetic groupings [22] and multiple locus variable number tandem repeat analysis (MLVA) provides more detailed assignments to genetic lineages and sub-lineages [22–24]. Within a single anthrax outbreak isolates will often group into a single or few MLVA defined genotypes. Within a MLVA type, more highly mutable genetic markers, single nucleotide repeats (SNRs), might be useful for tracing transmission pathways at fine spatial and temporal scales [25] and can detect *B. anthracis* diversity within outbreaks, and within individual carcass sites [26–29].

In this study, spatio-temporal data from a large anthrax outbreak in white-tailed deer, *Odocoileus virginanus,* in West Texas were analyzed to determine if spatio-temporal trends are supportive of proposed anthrax transmission mechanisms including contamination around carcass sites and fly mediated transmission. We also evaluated results of 25-marker MLVA (MLVA-25) and 4-marker SNR (SNR-4) analyses in the spatial and temporal contexts of the outbreak. Although the distribution of MLVA and SNR genotypes at regional to fine scales within outbreaks has been mapped [27, 29, 30] and epidemiological data has been linked to SNR analysis over a multi-year period [31], this study combined high resolution genotyping and spatiotemporal analyses to study a single outbreak in an anthrax system with a single browsing host species.

## Methods

### Study site

**Fig. 1:**
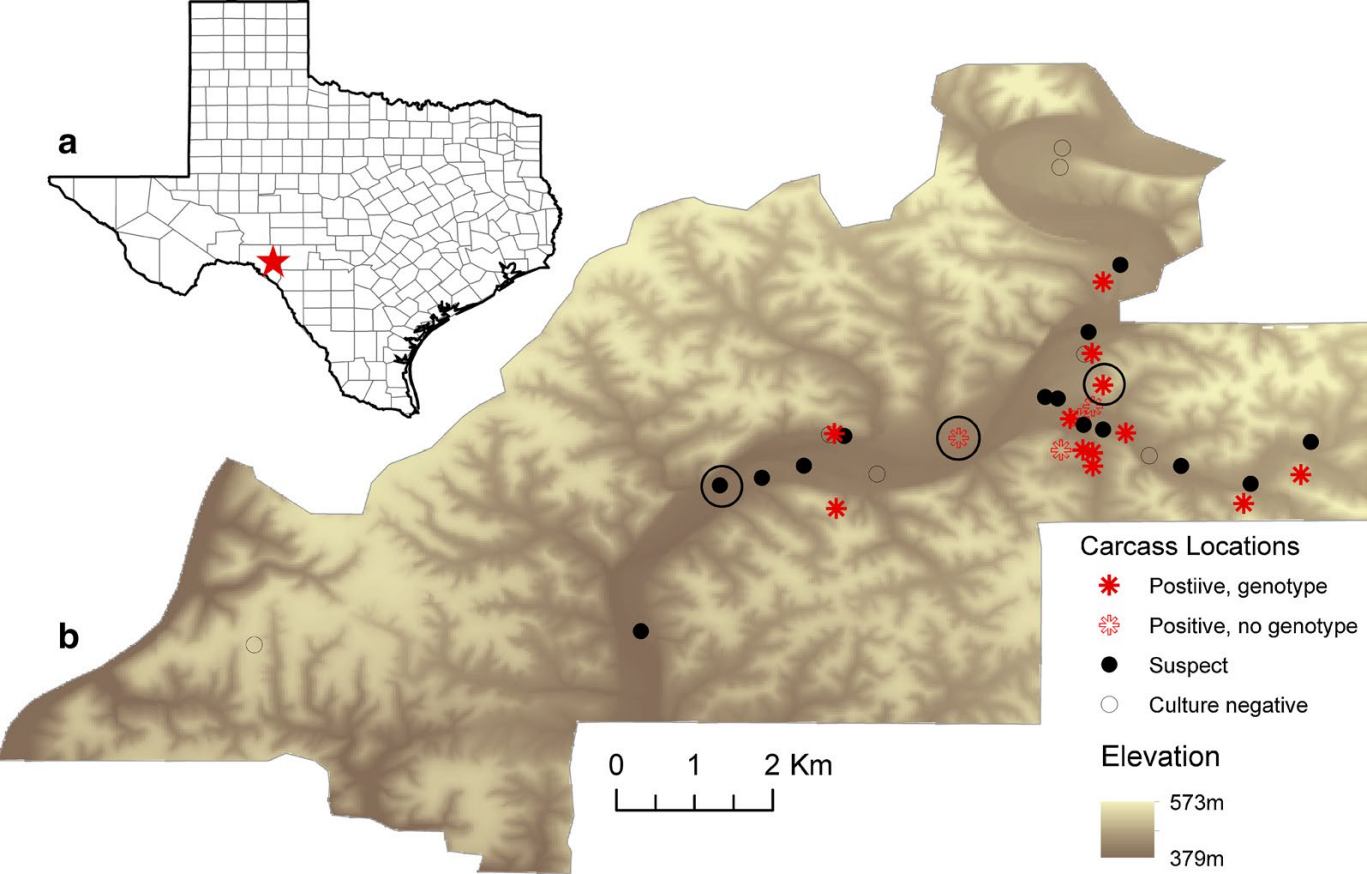
Location of study site and carcasses during the outbreak. **a** Location of study site (*red star*); **b** Locations of positive, negative and suspect carcasses shown over digital elevation map of the study area. Cases with dates of death on day 1 are circled. The original maps were produced in ArcGIS 10

The study site is a 7406 hectare ranch located ~80 km north of Del Rio, Texas along the Edwards Plateau in the anthrax enzootic zone (Fig. 1a). The eastern portion of the ranch is dominated by a branching, low lying dry riverbed having dense vegetation, whereas the western portion of the range has higher elevation, thinner soil and sparser vegetation. This ranch manages a herd of free range white-tailed deer, *Odocoileus virginanus,* and has an extensive history of anthrax, including large outbreaks in 2001 and 2005 [1]. Active surveillance has been conducted by ranch and research staff during the anthrax season, approximately May to October, since 2001; sporadic anthrax cases occur in most years [1]. Although the deer population is distributed widely across the ranch, carcasses have been found exclusively on the central and eastern portions of the ranch since surveillance began in 2001 [13]. During the anthrax season, white-tailed deer in this area primarily consume browse [16].

The 2005 epizootic had a mortality of at least 48 white-tailed deer, with a mortality estimate of ~3.7 % (95 % CI 2.7–4.9 %) of the herd. Daily movements of seven individual deer on this ranch were obtained by Blackburn [32] using daily acquired VHF-telemetry signals during the 2005 summer (July–August), including relocations during the outbreak period (September). For this study, we used the mean and maximum step lengths (distance between two consecutive relocations) to describe the movement potential of individual deer to examine the potential areas of infection during the outbreak [13] (movement metadata available online through Movebank [http://www.movebank.org]). Hematophagous fly density was also measured on the ranch during the outbreak season and is published elsewhere [13].

### Anthrax occurrence data

Data were derived from an acute anthrax outbreak during September 2005 [32]. All data was collected collaboratively with the ranch staff and permission of the land owner. Direct searching from the ground (including visual searches and seeking out carcasses by smell) and visualization of circling vultures, *Cathartes aura*, were used to locate carcasses. Carcass locations were recorded with handheld GPS units; date of death, gender, and age were estimated when possible. Forty-eight carcasses were identified during the outbreak period. Forty-one carcasses were geo-referenced; seven carcass sites were not GPS recorded at the time of carcass burning and we were unable to return to those sites during the response. Samples were obtained for microbiological testing from 17 animals; samples included ears, ribs, other bone fragments, and soil from under carcasses. After sampling, carcasses were disposed of by burning. Confirmed cases were carcasses from which samples were culture positive and from which *B. anthracis* DNA was amplified using polymerase chain reaction (PCR). Suspect cases were carcasses from which samples were not obtained but anthrax was suspected based on a typical saw-horse appearance to the carcass and evidence of shade seeking behavior. Seventeen clinically suspect carcasses were identified.

Anthrax was confirmed using classical microbiological methods and PCR as described in Blackburn et al. [17]. Briefly, after heat shocking (30 min at 70 °C) or suspension in 100 % ethanol for 1 h, samples were concentrated by centrifugation. The pellet was re-suspended in water and plated onto sheep blood agar and ACTSBA media. Gram staining and colony morphology were used for presumptive identification of *B. anthracis* and suspect colonies were sub-cultured for susceptibility to gamma phage lysis and for DNA extraction for PCR and genotyping. Carcasses with culture negative samples were excluded from these analyses.

### Spatial analysis

The outbreak was mapped in space and time in ArcGIS 10 (Environmental Systems Research Institute 2011. ArcGIS Desktop: Release 10. Redlands, CA, USA). Blackburn et al. [13] confirmed global clustering of carcasses during the outbreak using the average nearest neighbor index, which does not indicate the spatial scale of dependence. Here, we used the distance-based Ripley’s *K*-function [33] implemented in ArcGIS 10 to characterize the distance at which carcasses were clustered. The *K*-function is a second order analysis which identifies the spatial distance at which clustering occurs and is evaluated against a null distribution created using a Monte Carlo randomization [34]. The point of maximum difference between the observed and expected *K* values was used to indicate the distance at which cases cluster. The analysis was parameterized using bins of 40 m and a maximum distance of 1500 m, based on the minimum (40 m) and maximum (1259 m), daily movement of white-tailed deer as measured in 2005. All culture positive and suspect carcass locations were used for the *K*-function analysis.

### Spatio-temporal analyses

Trend analysis and the direction test were performed to explore for spatio-temporal patterns within the outbreak. Trend analysis is a global surface fitting procedure that tests for large scale, smooth changes in the data across the study area [35–37]. The procedure expresses the independent variable, *z*, here the date of death, as a polynomial function of the geographic coordinates, *x* and *y*, of carcass locations. A regression model is fit by a least squares method such that the sum of the squared deviations from the trend surface is minimized. First, second, and third polynomial trend surfaces were fitted using the Geostatistical Analyst Extension in ArcGIS 10 for carcasses with coordinates and estimated dates of death by day from the presumed first day.

**Fig. 2:**
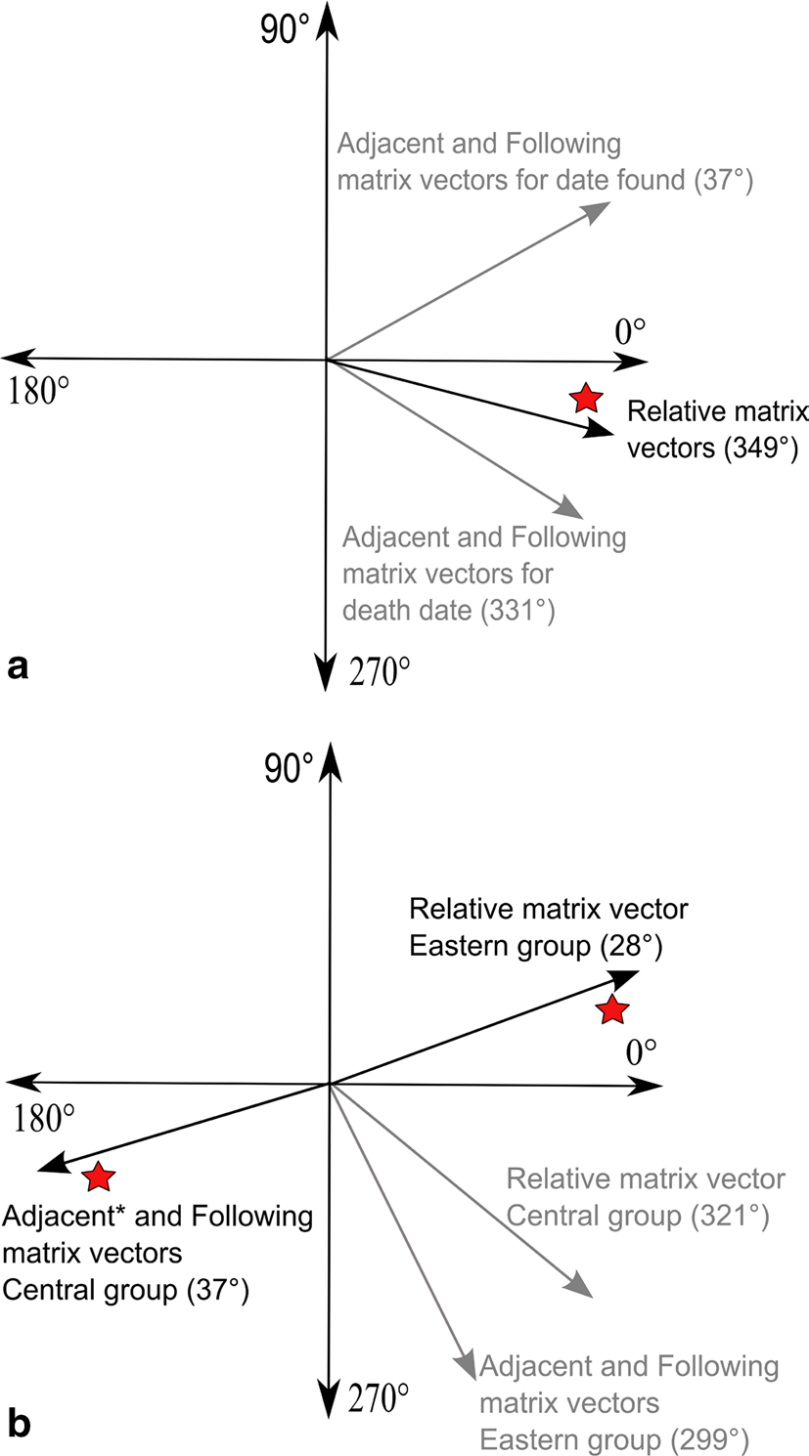
Spatial directions indicated by the direction test. East is at 0°, north is at 90°, west is 180° and south is 270°; **a** Using all carcass locations with dates of death and with date found; **b** Direction tests for eastern and central outbreak groups using date found as proxy for date of death. Vectors with significant magnitudes are indicated with a *red star*

The direction test uses retrospective individual case data to calculate the average direction in which cases advanced during an outbreak [38]. Details of the direction test can be found in Additional file 1: Protocol S1. Briefly, the test, which can be parameterized using one of three time connection matrices, outputs a directional vector for which east is 0 and north is 90 degrees (see Fig. 2) and a magnitude value of the vector ranging from 0 to 1. Each matrix was run using all carcasses with space and time data. Because of the distance between the locations of the carcasses estimated to have died on day 1 of the outbreak, the carcass locations were then divided into two groups under the hypothesis that there were two separate epizootic events. The direction tests were carried out separately for each group. The direction test was conducted in the ClusterSeer2 software package (http://www.biomedware.com).

### Genetic analysis

#### MLVA-25 genotyping

MLVA-25 genotyping was performed as described by Lista et al. [24], with minor changes in PCR chemistry and adaptations in primer labeling to perform analyses on the Applied Biosystems (ABI; Applied Biosystems, Foster City, CA, USA) instruments. Multiplex PCR products were diluted 1:25 in molecular grade water and 0.5 µL of the diluted multiplexes were mixed with 9.5 µL of a formamide/LIZ 1200 (ABI) size standard mixture and denatured.

#### SNR-4 genotyping

Samples were individually amplified for the four SNR loci described in Kenefic et al. [28]. The 10.0 µl PCR reactions were carried out with final concentrations of the following: 1.0 µL template DNA per reaction, 0.2 µM of each of the four forward and reverse primers, 1X PCR buffer, 0.5 U per reaction *pfu* Polymerase (Agilent technologies, Wilmington DE, USA), 3 mM MgCl_2_^*^, and 0.25 mM of each dNTP. The PCR products were pooled with a final dilution of HM-1, 2, and 6 at 1:20 and HM-13 at 1:10; 1.0 µL of the pooled products were mixed with 19.0 µL of a formamide/LIZ 500 (Applied Biosystems) size standard mixture and denatured.

Fragment sizing for MLVA-25 and SNR-4 was performed on an ABI 3730 (Applied Biosystems) and VNTR sizes were determined using GeneMapper™ software (Applied Biosystems). We examined genetic relationships between samples in the context of global representatives from Lista et al. [24] using Unweighted Pair Group Method with Arithmetic Averages (UPGMA) cluster analysis. Matrix distances were calculated in PAUP 4.0 (Sinauer Associates, Inc., Sunderland, MA, USA) and imported into MEGA 5 [39] in order to build phylogenetic trees based on MLVA-25. Data from SNR-4 analyses were evaluated using differences in base pair repeats between isolates grouping within MLVA-25 genotypes and UPGMA.

## Results

### Outbreak description

Thirty positive or suspect cases were georeferenced and had gender determined; 18 (60 %) were male (Additional file 2: Table S1). A date of death was determined for 20 (67 %). *Bacillus anthracis* was cultured from 16 of 17 carcasses from which samples were obtained A presumptive *B. anthracis* isolate was recovered from the additional carcass, but failed to genotype as *B. anthracis* using MLVA-25 typing. Upon subsequent testing, it was negative on the confirmatory *plcR* SNP assay [40] and excluded. The first positive carcasses were found September 6th with an estimated September 4th date of death, and the last carcass was found on September 28th. Locations of carcasses are shown plotted on a 10 m digital elevation map (DEM) of the study area in Fig. 1b. Three cases were estimated to have died on the presumed first day of the outbreak, September 4th, are circled in Fig. 1b.

### Spatial analysis

**Fig. 3:**
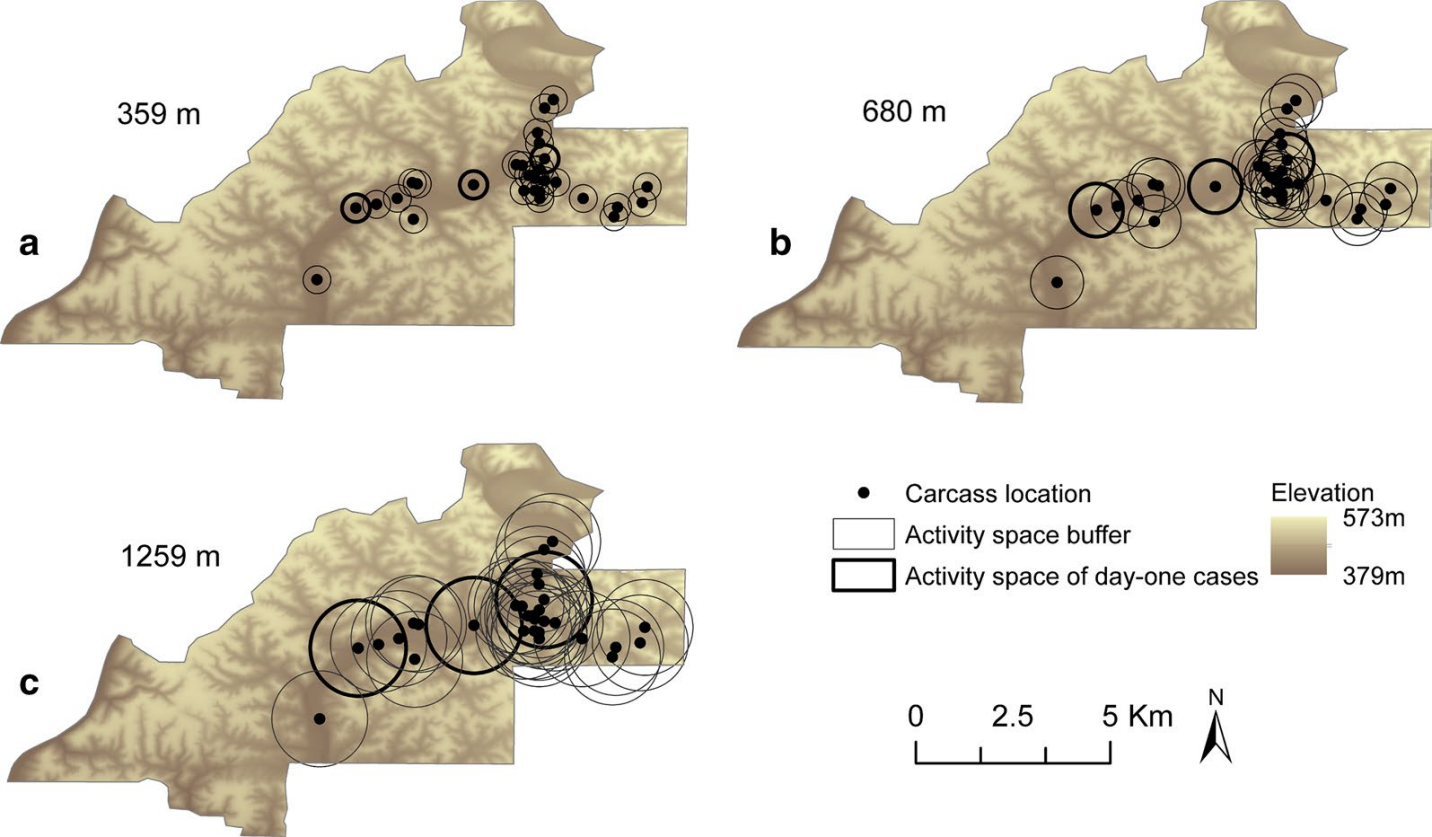
Carcass locations and deer movement estimates; **a** mean daily deer movements; **b** K-function-defined cluster distance; **c** maximum daily deer movement. Maps produced in ArcGIS 10

The *K*-function analysis identified the maximum clustering of cases at a distance of 680 m (Additional file 3: Fig. S1). The potential overlap of deer movement with nearby carcass locations using mean and maximum movement estimates of 359 m and 1259 m, respectively [13], and the K-function distance of 680 m, is shown in Fig. 3.

### Spatio-temporal analysis

**Fig. 4:**
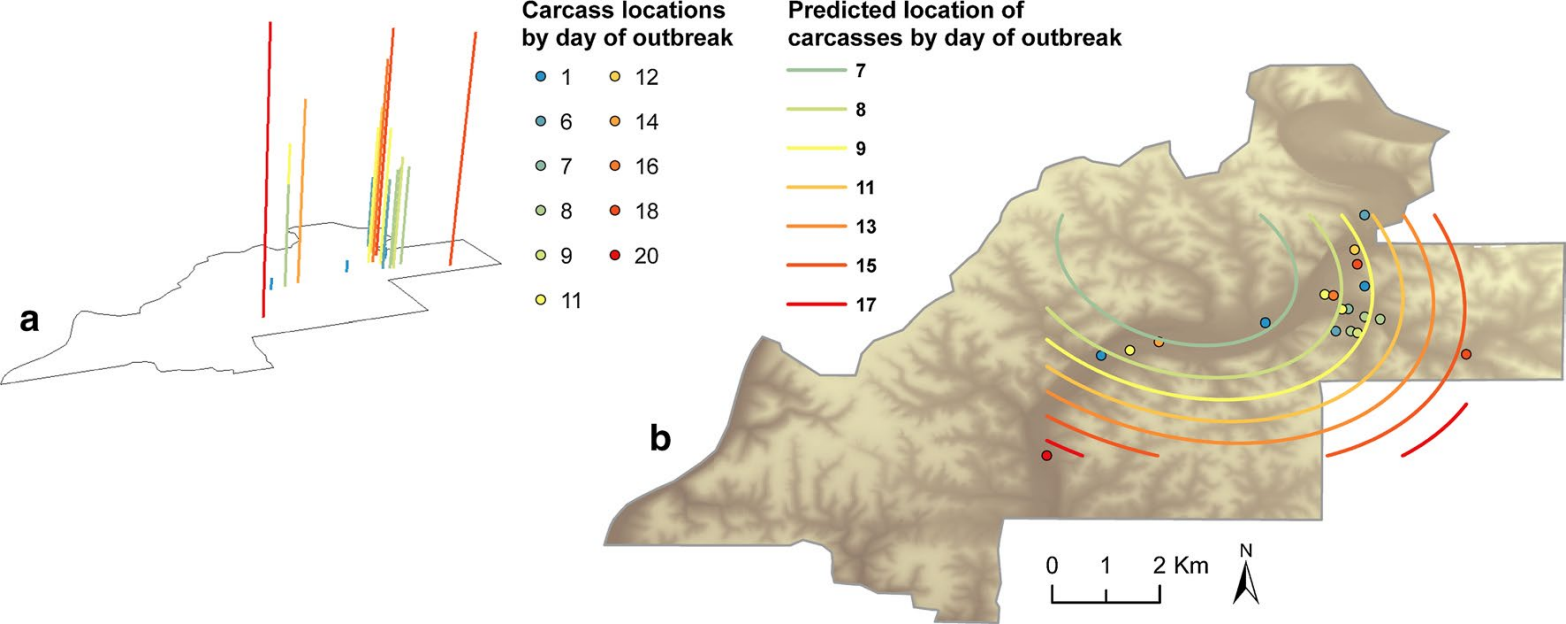
Three-dimensional representation of the outbreak and trend surface; **a** 3-dimensional plot of 20 carcass locations with geographic and temporal data; **b** Predicted carcass locations as interpolated by a trend-surface analysis model using all cases with geographic and temporal data. Maps produced in ArcGIS 10

Figure 4a illustrates the locations of the 20 cases with data in space and time; vertical bar height corresponds to time between the estimated start of the epizootic and date of death. Results of second polynomial trend analysis suggested new cases spread outward during the outbreak with an overall trend towards the southeast and southwest (Fig. 4b).

The result of the relative matrix direction test using the 20 cases with date of death estimates was a vector of 349.41 degrees (east being 0) (Fig. 2a), indicating an east—southeast advance of cases, although the magnitude of the vector, 0.0299, was not significantly different from the null hypothesis (*p* value = 0.7220). Adjacent and following time connection matrices produced similar results (Table 1). To evaluate the effect of sample size and data collection bias, the tests were repeated using all 30 cases with both geographic coordinates and at minimum a date found. Date found was used in lieu of date of death when the latter was unknown. The average angle of the relative matrix vector was then 347.51 with a significant concentration of 0.1406 (p = 0.0010). Applying the adjacent and following matrices to this dataset changed the average vector angles to 36.99 (both matrices) with non-significant magnitudes of 0.6060 and 0.7600, respectively. However, when the group of 15 cases in the eastern portion of the ranch was considered separately from the group of five located near the center of the ranch, the results suggested a different set of patterns (Fig. 2b). In the eastern outbreak group, the relative direction test resulted in a vector of 27.66 degrees, or an east-northeast direction, with a magnitude of 0.1704 (p-value 0.0020) and the following and adjacent matrices had low magnitude southeast vectors. The central outbreak group had a relative matrix result of an east-southeast vector of moderate magnitude which approached significance (321.31, 0.3298, p = 0.0760), and matrices measuring shorter term patterns shifted the directionality to the west (Table 1).

**Table 1:** Results of the direction tests using each of three matrices for connecting cases

Matrix	Death date			Date found for unknown death dates		
	Average angle	Concentration	P-value	Average angle	Concentration	P-value
Relative	349.41	0.0299	0.7220	347.51	0.1406	*0.0010*
Adjacent	331.37	0.2187	0.2700	36.99	0.1225	0.6060
Following	331.37	0.2187	0.5320	36.99	0.1225	0.7600

Matrix	Eastern outbreak group			Central outbreak group		
	Average angle	Concentration	p-value	Average Angle	Concentration	p-value
Relative	27.6607	0.17038	*0.0020*	321.31	0.3298	0.0760
Adjacent	298.542	0.0986	0.7970	194.002	0.6656	*0.0460*
Following	298.542	0.0986	0.9010	194.002	0.66581	0.1890

Significant p-values are shown in italic. The results of the direction tests are illustrated graphically in Fig. 2

### Genetic analysis

**Fig. 5:**
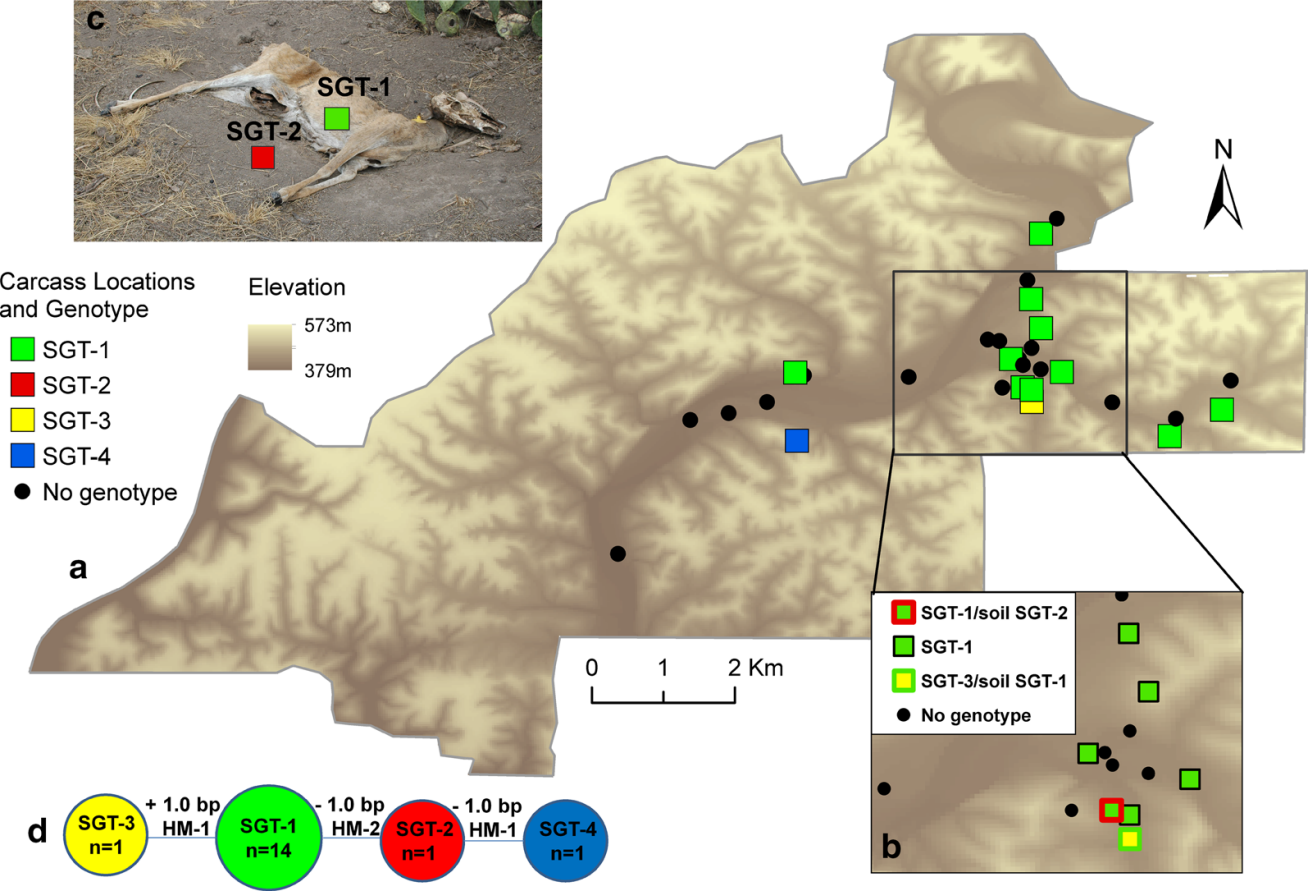
Results of SNR genotyping; **a** Carcass locations and SNR genotypes (SGT); **b** Detail of the high transmission area in which 2 SGTs were found at each of two carcass sites; **c** A typical carcass found during the epizootic and sampling scheme through which diverse SGTs were collected; **d** Genetic relationships among the 4 SGTs. Maps produced in ArcGIS 10

Of the sixteen *B. anthracis* positive samples, 14 were from carcasses and two were from soil adjacent to carcasses. All isolates were identical on MLVA-25 and grouped into the A4 (Vollum-like) cluster using the MLVA-25 nomenclature and the genotype 57 group using the Lista et al. [24] groupings (Additional file 4: Fig. S2). SNR analysis of the 16 isolates yielded four subgenotypes (SGT; Table 2). The most frequently identified SGT among the isolates available in this outbreak designated SGT-1. The isolate associated with the earliest date of death was SGT-1. Two different SNR genotypes were isolated from a carcass and adjacent soil at each of two carcass sites. SGT-2 differed from SGT-1 by a subtraction of one base pair at HM-2 and was isolated from the soil adjacent to a carcass from which SGT-1 was isolated. SGT-3 differed from SGT-1 by addition of one base pair at HM-1 and was isolated from a carcass adjacent to a soil sample which yielded a SGT-1 isolate. SGT 4, isolated from a carcass in the central portion of the ranch, differed from SGT-1 at markers HM-1 and HM-2. SGT-3 and SGT-4 were both isolated from animals found on September 14th; date of death was not estimated for these two cases. Figure 5 shows locations of all SGTs as well as the sampling location for carcass and soil isolates.

**Table 2:** Allele sizes used to characterize sub-genotypes of *Bacillus anthracis* recovered from white-tailed deer during an anthrax outbreak

Subgenotype	Allele size (number of base pairs at locus)				
	HM1	HM2	HM6	HM13	No. isolates
SGT1	84	106	90	118	14
SGT2	84	[105]	90	118	1
SGT3	[85]	106	90	118	1
SGT4	[83]	[105]	90	118	1

Brackets reflect base pair differences between subgenotypes

## Discussion

The ecology of anthrax, like many zoonoses, particularly those with environmentally maintained pathogens and indirect transmission, is complex. The ecology of the primary host species, seasonal variations in host resource use, predation pressures, and insect vector populations, as examples, shape spatial and temporal variations in anthrax dynamics [41, 42]. Outbreaks can be associated with climatic events [1] and evidence suggests local pathogen population genetics can be associated with soil characteristics [5] or regional climatic variables [43]. Because of this complexity, fine scale analyses of anthrax outbreaks are essential to characterizing local disease ecology and planning prevention and control. In this study a single outbreak in West Texas was analyzed. This study had two objectives, one, to explore spatial and spatio-temporal characteristics of the outbreak and, two, to place high resolution genetic analysis in the context of the spatio-temporal findings.

The *K*-function analysis indicated carcasses tended to be clustered at a distance of approximately 680 meters. This distance is well within the daily activity space of deer in this herd as measured during the outbreak period [13]. In addition to this spatial clustering, carcasses were concentrated in the low lying dry riverbeds which are a primary landscape feature of the eastern portion of the ranch; this was true even though census data showed not only that the deer population was distributed across the ranch, but deer density was higher in the western region [13]. The clustering distance has implications for transmission. Shrub-like vegetation is denser in the riverbeds than at higher elevations; during the summer anthrax season, deer in Texas primarily consume leaves (browse) [44] facilitating exposure to anthrax spores potentially deposited on browse by necrophagous flies. Deer could have encountered carcass sites within their home ranges and been exposed to freshly contaminated browse [16], or to contaminated biting flies [13, 14]. Spatial overlap between biting fly densities and carcass locations was confirmed during this outbreak, in particular in the east [13].

An exception to the overlapping activity space/home range scenario is the case occurring on day 20 to the western most reach of the epizootic. This carcass, which was located in a riverbed, could have resulted from an independent exposure to an environmental source; the deep soil conditions in the riverbeds are thought to promote and sustain *B. anthracis* persistence [2–4], possibly providing multiple spatially dispersed opportunities for new infections.

Alternatively, there was overlap with neighboring cases’ home ranges at the maximum step length. The relative isolation of this carcass location may also be explained by missed carcasses, higher than average movement of that animal, or an insect vector. This location underscores the potential challenges of characterizing anthrax burdens from mortality events. Recent modeling work in the Etosha National Park, Namibia, indicated approximately 3.8 times more zebra mortalities occurred than carcasses were sighted [45]. Although these models were specific to zebras in the Etosha ecosystem, the findings emphasize actual mortality in an outbreak is higher than the numbers of carcasses identified. This is likely true with deer outbreaks in Texas, where the landscape is vast and the deer carcasses small.

The second polynomial trend analysis suggested transmission processes expanded the outbreak spatially, particularly towards the southeast and to a lesser extent to the southwest. Given evidence showing individuals did not travel long distances as a result of the outbreak, transmission mechanisms driven by insects or contact with contaminated browse at carcass sites, combined with a concentration of these factors in riverbeds, could have produced this expansion pattern. A second approach to detecting significant patterns in the outbreak in space and time was to evaluate the directionality of the outbreak. The direction test using the relative time connection matrix and cases with estimated dates of death indicated the chain of infections trended to the east-southeast, and results of the adjacent and following matrices were similar. Both the trend analysis and the direction test indicated cases advanced in a southeast direction towards an area of high SNR-4 diversity. When the direction test was repeated using all carcasses with geographic coordinates, however, shorter term processes trended to the east-northeast. These directionalities roughly paralleled the dry river beds. Multiple carcasses with dates of death on day zero of the outbreak suggest a widespread source of exposure could have initiated this outbreak. In this ecosystem, there is evidence of increased spore availability following extreme weather events [1]. Subsequent outward, directional spread of new infections following these environmental exposures is supportive of an environmentally mediated transmission pathways, such as necrophagous flies, via contaminated browse, or hematophagous flies. When we divided the carcass locations into two geographic groupings under the possibility of two separate outbreak events, the two groups had different spatio-temporal vectors using matrices measuring short term processes. Mechanisms of transmission may differ between these areas due to variations in micro-scale climate and ecology, animal behavior, or fly population dynamics; in the east, the direction test results were consistent with spread in the area where biting fly densities were highest [13]. We cannot, however, rule out missed carcasses or widespread spore availability.

The finding of identical adjacent and following vector angles suggests that transmission occurs primarily through processes acting on shorter temporal scales, consistent with findings that survival of *B. anthracis* spores is temporally limited by conditions common to summer months in this region (i.e., ultra violet light and high temperatures) [46] and more recent evidence of pathogen survival being limited in fly feces or emesis on leaves in the region [17]. We note that use of different time connection matrices resulted in different vector directions; however, matrix selection is rarely explicitly addressed in the literature making comparisons difficult.

We evaluated whether the addition of genotyping to the spatial analyses would improve our ability to trace transmission across the landscape. All isolates were identified as a single MLVA-25 genotype. The earliest identified and most common SNR genotype in the outbreak was SGT-1; three other genotypes represented polymorphisms between and within carcass sites. Carcasses associated with genotypes SGT-2, 3, and 4 were found mid-way through the epizootic period. In the context of the overall spatial and temporal course of the outbreak, two of the three sites with unique SGTs were in an area in which a number of carcasses were in relatively close spatial and temporal proximity and, relative to the day one cases, were located along the vector for the chain of infection. While the spatial and temporal distribution of SGTs did not present a spatio-temporally linear transmission chain, the spread of new of cases with increasing diversity are again supportive of fly mediated transmission following an environmental exposure.

The diversity between and within carcass locations in this report concur with previous reports of SNR genotypes appearing spatially and temporally dispersed in another large (multiple population) epizootic [29] and of multiple SNR types within intensively sampled carcass sites [30]. These findings present challenges to interpreting fine scale *B. anthracis* genetic diversity in the context of a single outbreak or epizootic. SNR diversity is determined by sporadic and outbreak incidence rates, in vivo mutation rates, relative selection within hosts and micro- environments, level of bacteremia, and spore survival. For *B. anthracis* in natural systems these parameters remain poorly understood; therefore, the SNR mutational changes occurring during an outbreak cannot yet be placed in the proper evolutionary and probabilistic context and differentiated from sampling artifact [31]. In Namibia, MLVA genotyping of isolates collected over multiple outbreak years showed few MLVA defined genotypes persisted across time, but one large outbreak was dominated by a MLVA genotype subsequently found only sporadically [31]. Genotyping of isolates from this ranch over multiple years identified only one MLVA type to date [47]; this limited diversity may in part be due to intensive efforts by ranch personnel to destroy carcasses, limiting the opportunity for diversity.

The findings of this study are potentially limited by the small proportion of total carcasses identified having complete data. During efforts to control the outbreak, multiple carcasses were burned before samples and data could be collected, and burned remains were consistently culture negative. Moving forward, systematic and intensive sampling of environmental samples before and after outbreaks will be critical to gain a comprehensive understanding of the distribution and genetic diversity of *B. anthracis* on the landscape. These efforts should be part of intensive control efforts and require timely cooperation of ranch managers, veterinarians, and researchers.

## Conclusions

Among white-tailed deer in West Texas, anthrax transmission events occurred within limited spatial areas and carcass locations were within activity spaces of subsequent cases, indicating interaction of animals with carcass sites during outbreaks is essential to anthrax outbreak dynamics in this landscape. The pathology of infection during a West Texas outbreak is not known, making it impossible to differentiate between biting flies, which would lead to cutaneous infection [14], or ingestion of browse contaminated by necrophagous flies. These results provide spatial and temporal support for the role of hematophagous or necrophagous flies in anthrax outbreaks, highlighting the need for better characterization of infection and confirmation of transmission by either group of flies. These findings also emphasize the need for active surveillance and prompt cleanup of anthrax carcasses to control anthrax both during outbreaks and between seasons.

## Additional files

10.1186/s12898-015-0054-8 Methodological description of the Direction test.
10.1186/s12898-015-0054-8 A table of deer deaths from anthrax during the 2005 outbreak including estimated date of death, gender, and disease status.
10.1186/s12898-015-0054-8 Ripley’s K plot of deer carcass locations from the 2005 outbreak. Blue line indicates the difference between the observed and expected K values. The black vertical line identifies the maximized clustering distance as the value with the greatest difference between observed and expected values.
10.1186/s12898-015-0054-8 Phylogenetic tree placing the 2005 outbreak in the global context of *Bacillus anthracis* diversity based on the 25 marker MLVA system. The outbreak strains are indicated in blue. Dominant global lineages are identified in red.

## Abbreviations

CanSNP: canonical single nucleotide polymorphism
DEM: digital elevation model
MLVA: multiple locus variable number tandem repeat analysis
PCR: polymerase chain reaction
SNP: single nucleotide polymorphism
SNR: single nucleotide repeat
UPGMA: unweighted pair group method with arithmetic averages
VNTR: variable number tandem repeat
