# Supplementary material for: Spatio-temporal patterns of an anthrax outbreak in white-tailed deer, Odocoileus virginanus, and associated genetic diversity of Bacillus anthracis
Source: BMC Ecol. 2015 Dec 15;15:23. doi: 10.1186/s12898-015-0054-8 (PMC4681179; doi:10.1186/s12898-015-0054-8)
Supplement: Supplementary file 1 — 10.1186/s12898-015-0054-8 Methodological description of the Direction test. [file 12898_2015_54_MOESM1_ESM.docx]

**Supplementary Protocol S1. The direction test.**

**Direction test**

The direction test uses retrospective individual case data to calculate the average direction in which cases advance during an outbreak[^1^](#_ENREF_1). The output is a directional vector for which east is 0, north is 90 degrees, west is 180 degrees and south is 270 degrees, and a magnitude value of the vector ranging from 0 to 1. The magnitude, also called the angular concentration, represents the variance in the angles of the cases contributing to the vector. A magnitude approaching 1 indicates less variance in the angles and therefore a more concentrated directionality, whereas a magnitude of the vector approaching 0 indicates the outbreak pattern is more dispersed. Significance of the test is evaluated with a randomization procedure equivalent to holding the case locations fixed and varying their temporal occurrence and repeating the test to generate a null distribution against which the test data can be compared. Cases may be connected in time in one of three time connection matrices. In a ‘relative’ time matrix, each case is connected to all of the subsequent cases and is best for understanding processes occurring over longer time spans. The ‘adjacent’ matrix connects cases only to their nearest neighbors in time and is best for processes occurring over shorter durations. The ‘following’ matrix connects each case only to the one immediately subsequent and is appropriate for tracing the average direction of transmission processes. Each of the time connection matrices was attempted to explore the scale of possible processes driving transmission.

1 Jacquez, G. M. Statistical software for the clustering of health events. *Statistics in medicine* **15**, 951-952 (1996).
