# Supplementary figures and images for: Spatio-temporal patterns of an anthrax outbreak in white-tailed deer, Odocoileus virginanus, and associated genetic diversity of Bacillus anthracis
Source: BMC Ecol. 2015 Dec 15;15:23. doi: 10.1186/s12898-015-0054-8 (PMC4681179; doi:10.1186/s12898-015-0054-8)

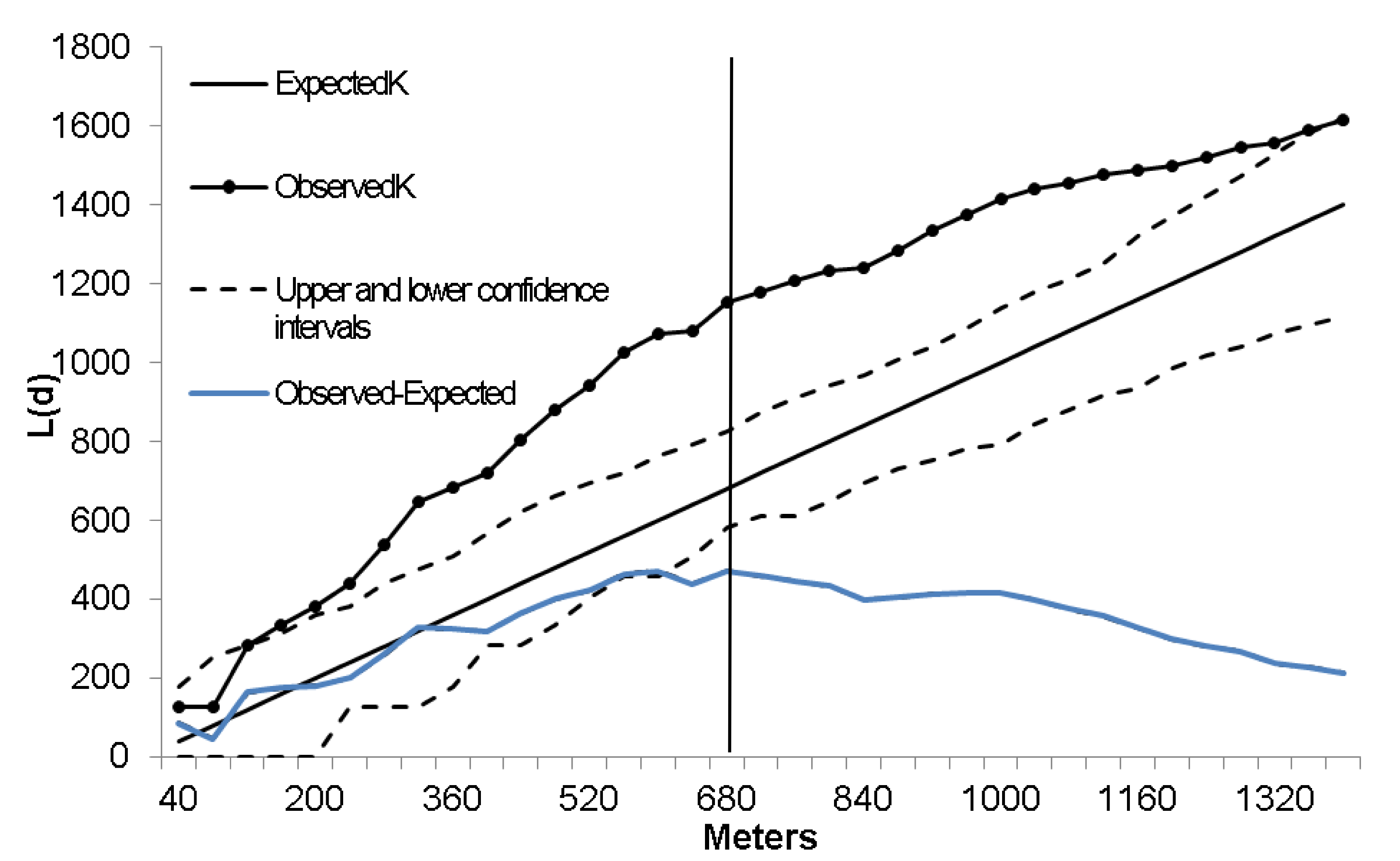

Supplement: Supplementary file 3 — 10.1186/s12898-015-0054-8 Ripley’s K plot of deer carcass locations from the 2005 outbreak. Blue line indicates the difference between the observed and expected K values. The black vertical line identifies the maximized clustering distance as the value with the greatest difference between observed and expected values. [file 12898_2015_54_MOESM3_ESM.tif]

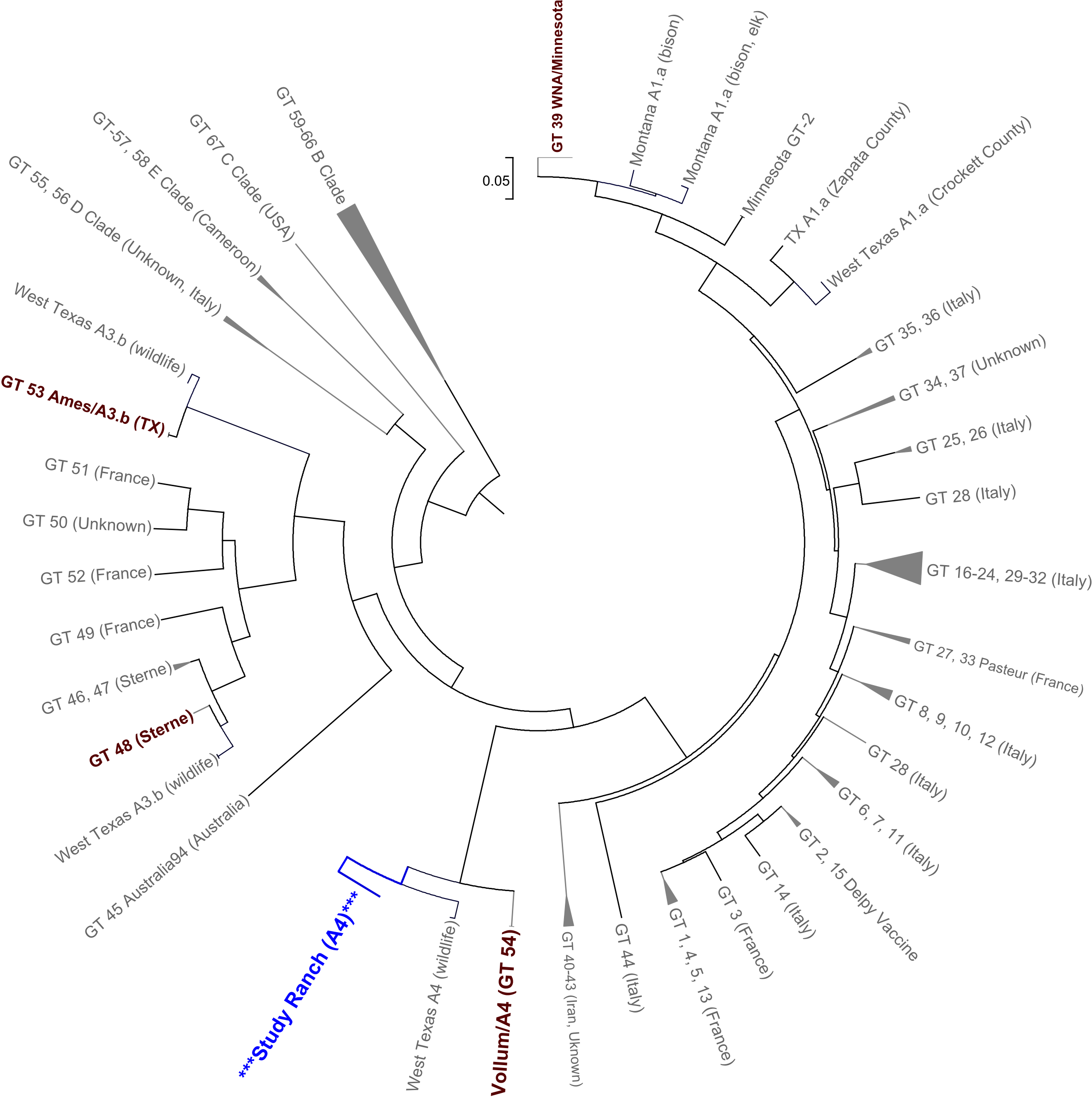

Supplement: Supplementary file 4 — 10.1186/s12898-015-0054-8 Phylogenetic tree placing the 2005 outbreak in the global context of Bacillus anthracis diversity based on the 25 marker MLVA system. The outbreak strains are indicated in blue. Dominant global lineages are identified in red. [file 12898_2015_54_MOESM4_ESM.tif]
